# Genetic Surveillance of SARS-CoV-2 M^pro^ Reveals High Sequence and Structural Conservation Prior to the Introduction of Protease Inhibitor Paxlovid

**DOI:** 10.1128/mbio.00869-22

**Published:** 2022-07-13

**Authors:** Jonathan T. Lee, Qingyi Yang, Alexey Gribenko, B. Scott Perrin, Yuao Zhu, Rhonda Cardin, Paul A. Liberator, Annaliesa S. Anderson, Li Hao

**Affiliations:** a Vaccine Research & Development, Pfizer Inc., Pearl River, New York, USA; b Medicine Design, Worldwide Research & Development, Pfizer Inc., Cambridge, Massachusetts, USA; c Digital R&D Creation Center, Pfizer Digital, Pfizer Inc., Pearl River, New York, USA; Icahn School of Medicine at Mount Sinai

**Keywords:** surveillance, SARS-CoV-2, M^pro^, 3CL^pro^, mutation, purifying selection, nirmatrelvir, Paxlovid

## Abstract

Severe acute respiratory syndrome coronavirus 2 (SARS-CoV-2) continues to represent a global health emergency as a highly transmissible, airborne virus. An important coronaviral drug target for treatment of COVID-19 is the conserved main protease (M^pro^). Nirmatrelvir is a potent M^pro^ inhibitor and the antiviral component of Paxlovid. The significant viral sequencing effort during the ongoing COVID-19 pandemic represented a unique opportunity to assess potential nirmatrelvir escape mutations from emerging variants of SARS-CoV-2. To establish the baseline mutational landscape of M^pro^ prior to the introduction of M^pro^ inhibitors, M^pro^ sequences and its cleavage junction regions were retrieved from ~4,892,000 high-quality SARS-CoV-2 genomes in the open-access Global Initiative on Sharing Avian Influenza Data (GISAID) database. Any mutations identified from comparison to the reference sequence (Wuhan-Hu-1) were catalogued and analyzed. Mutations at sites key to nirmatrelvir binding and protease functionality (e.g., dimerization sites) were still rare. Structural comparison of M^pro^ also showed conservation of key nirmatrelvir contact residues across the extended *Coronaviridae* family (α-, β-, and γ-coronaviruses). Additionally, we showed that over time, the SARS-CoV-2 M^pro^ enzyme remained under purifying selection and was highly conserved relative to the spike protein. Now, with the emergency use authorization (EUA) of Paxlovid and its expected widespread use across the globe, it is essential to continue large-scale genomic surveillance of SARS-CoV-2 M^pro^ evolution. This study establishes a robust analysis framework for monitoring emergent mutations in millions of virus isolates, with the goal of identifying potential resistance to present and future SARS-CoV-2 antivirals.

## INTRODUCTION

The causative agent of coronavirus disease 2019 (COVID-19) was identified as a novel coronavirus (CoV) (1), later named severe acute respiratory syndrome coronavirus 2 (SARS-CoV-2), with close genetic and clinical resemblance to the 2002 SARS virus (SARS-CoV) (2, 3). SARS-CoV-2 shares the core features of all CoVs, including a large positive-stranded RNA genome (26 to 32 kb), the spike (S), envelope (E), membrane (M), and nucleocapsid (N) structural proteins, as well as two conserved viral proteases: the main protease (M^pro^), also known as 3-chymotrypsin-like cysteine protease (3CL^pro^), and papain-like protease (PL^pro^) (4). These enzymes digest two large polyproteins (pp1a and pp1ab) at multiple junctions to generate a series of proteins critical for virus replication and transcription, including the RNA-dependent RNA polymerase (RdRp), helicase, and the M^pro^ protein itself (5). M^pro^ is encoded by open reading frame 1 (ORF1) as nontructural protein 5 (Nsp5) and cleaves the polyproteins at 11 sites to release Nsp4 to Nsp16, making M^pro^ an essential protein for the CoV life cycle (6).

Since the onset of the COVID-19 pandemic in 2020, SARS-CoV-2 variants have rapidly emerged worldwide, raising concern for the effectiveness of currently available vaccines and neutralizing monoclonal antibodies (MAbs) targeting the S protein. As of March 2022, the World Health Organization (WHO) has identified five major variants of concern (VOCs): B.1.1.7 (Alpha, α), B.1.351 (Beta, β), P.1 (Gamma, γ), B.1.617.2 (Delta, Δ), and most recently, B.1.1.529 (Omicron, o) (7). Characterization of emergent variants has centered on the number and location of mutations in the S protein trimer (8). Omicron, specifically, contains several signature mutations in the S protein that enable the variant to escape immunity from previous infection or vaccination (9), making it unlikely that each of the approved MAbs will maintain clinical efficacy against this VOC (10). To date, the only approved or authorized non-MAb therapeutics for COVID-19 are small-molecule antivirals: remdesivir and molnupiravir, both RdRp inhibitors originally developed for different RNA viruses, and Paxlovid, whose antiviral component, nirmatrelvir, a CoV M^pro^ inhibitor, is coadministered with ritonavir. Remdesivir is administered intravenously, while molnupiravir and Paxlovid are orally bioavailable.

Nirmatrelvir is an active site inhibitor of the SARS-CoV-2 M^pro^ that exhibits *in vitro* antiviral activity across the *Coronaviridae* family, demonstrating potent inhibition of the M^pro^ from all other β-coronaviruses (β-CoVs) and α-coronaviruses (α-CoVs) known to infect humans (11). Active sites of M^pro^ are largely conserved among β-CoVs. The SARS-CoV-2 M^pro^ amino acid sequence shares 96% identity with that of SARS-CoV, with differences at 12 residues between the two viruses (12). The critical amino acid residues involved in enzyme-inhibitor binding interactions are also particularly well conserved within this family of viruses (13). Its essential functional importance in virus replication, together with the absence of closely related homologues in humans (14), identify the CoV M^pro^ as an attractive antiviral drug target (11, 15). Indeed, Paxlovid was granted emergency use authorization (EUA) from the FDA in December 2021, after positive results in the phase 2/3 Evaluation of Protease Inhibition for COVID-19 in High-Risk Patients (EPIC-HR) trial (16).

In such a rapidly evolving pandemic, it is important to monitor resistance of emerging variants to compounds targeting critical viral proteins, including M^pro^. Among the many unprecedented aspects of the ongoing COVID-19 pandemic is an intense phylogenetic surveillance of the virus in the human population. The genome sequences of millions of SARS-CoV-2 isolates have been determined and deposited into the GISAID database (17) since January 10, 2020. The accessibility of real-world sequences from the expansive GISAID data set has enabled a global, collaborative effort by scientists to track emerging lineages, identify signature escape mutations, and classify new variants in real time (18). To our knowledge, a comprehensive genomic surveillance of mutations in SARS-CoV-2 nonstructural proteins is limited to the RdRp (19, 20). Large-scale genetic surveillance of the M^pro^ enzyme from circulating SARS-CoV-2 variants has yet to be reported.

In the present study, we built a workflow to monitor the evolution of M^pro^ and the emergence of potential escape mutations in millions of SARS-CoV-2 genomes obtained from GISAID. We address the suitability of M^pro^ as a drug target for COVID-19 by evaluating polymorphisms at M^pro^ dimerization and substrate cleavage sites, in addition to key contact residues with the selective inhibitor nirmatrelvir, and thus provide a baseline understanding of M^pro^ diversity prior to the widespread use of Paxlovid.

## RESULTS

### Structural and sequence conservation of M^pro^ from different CoVs.

**FIG 1:**
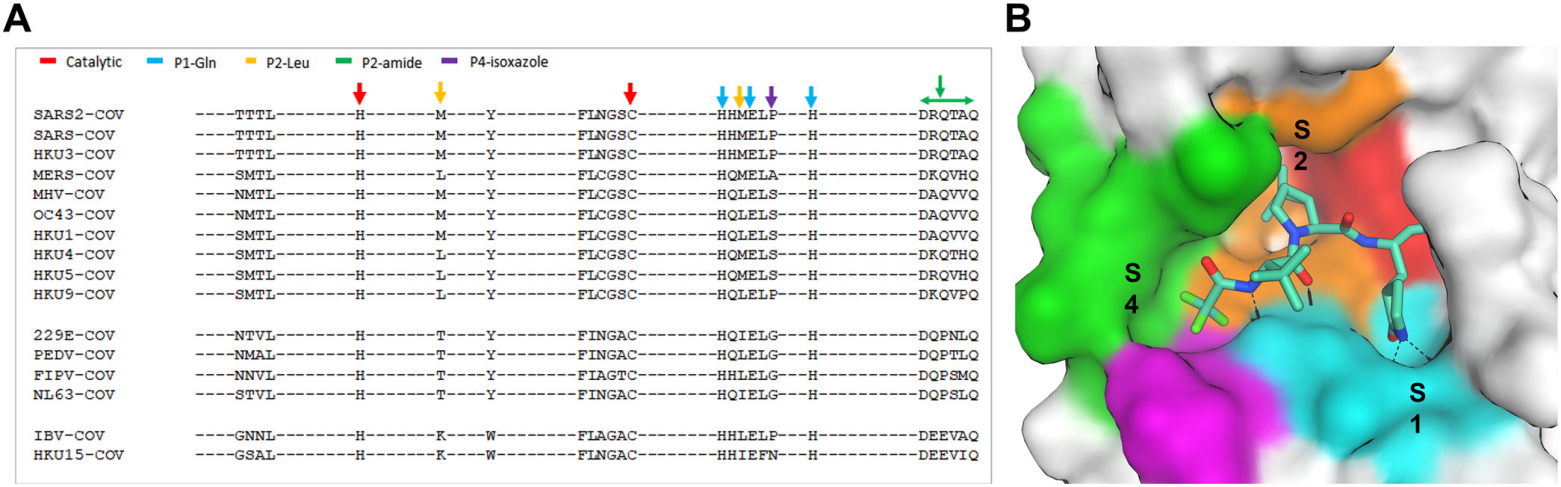
Active site conservation of coronavirus (CoV) main proteases. (A) Sequence alignment of the 26 binding site amino acids. The key amino acids with relative positions (P) are indicated by color-coded arrows based on their interaction with the inhibitor, nirmatrelvir. (B) severe acute respiratory syndrome coronavirus 2 (SARS-CoV-2) M^pro^-binding pocket of nirmatrelvir. The pocket surface is colored based on the inhibitor’s interaction shown in panel A.

**FIG 2:**
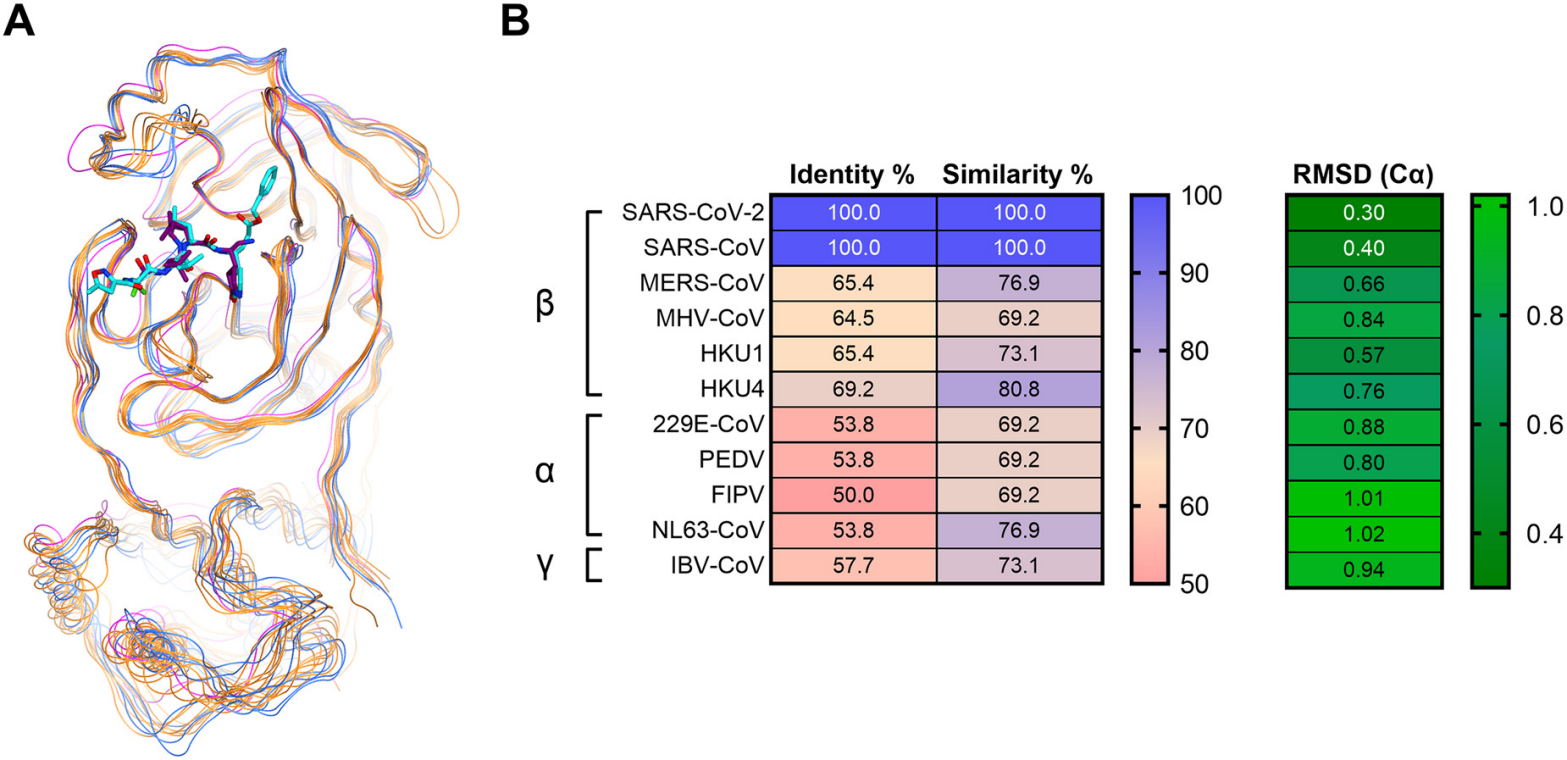
Comparison of structure and sequence identity across 12 CoV main proteases. (A) Superposition of 12 CoV main proteases based on the 26-amino acid backbone heavy atoms at the active site. The proteases are represented by colored lines, with β-CoV proteases in yellow, α-CoV proteases in blue, and γ-CoV protease in magenta. The complete list of CoV proteases can be found in Table S1. (B) Percent sequence identity, similarity, and root mean square deviation (RMSD) (Cα, alpha-Carbon) of 26 amino acids at the nirmatrelvir-binding site for β-CoVs, α-CoVs, and IBV-CoV (γ-CoV). Identity and similarity values range from 50 to 100, and RMSD (Cα) values range from 0.30 to 1.02 in their respective color-mapping scales.

Nirmatrelvir was previously demonstrated to have robust pan-CoV antiviral activity (11). To further investigate the conservation of M^pro^ across the extended *Coronaviridae* family, we examined the conservation of M^pro^ active sites from α-CoVs (*n = *4), β-CoVs (*n = *7, including SARS-CoV-2), and γ-coronaviruses (γ-CoVs) (*n = *1) from a structural perspective. The active site amino acid sequence (Fig. 1) and conformational differences (Fig. 2) of multiple M^pro^ enzymes were compared among the selected Protein Data Bank structures (Table S1). Twenty-six amino acids were selected as active site residues because they have at least one heavy atom within 4.5 Å of the common ligand PRD_002214. PRD_002214 is a Michael acceptor-based peptidomimetic inhibitor, known as N3, developed previously to target M^pro^ from multiple CoVs (21–24). Since then, this inhibitor has been used in broad CoV M^pro^ enzymatic and cocrystallographic studies, including the first reported SARS-CoV-2 M^pro^ crystallographic structure (25).

10.1128/mbio.00869-22.3 TABLE S1 List of CoV protease structures in the homology analysis. Download Table S1, PDF file, 0.03 MB. Copyright © 2022 Lee et al. 2022 Lee et al. https://creativecommons.org/licenses/by/4.0/ This content is distributed under the terms of the Creative Commons Attribution 4.0 International license.

The sequence homology comparison of these 26 amino acid residues in M^pro^ across different CoVs is shown in Fig. 1A The key interaction amino acids are also indicated by arrows colored by their location at the binding site (Fig. 1B). The catalytic site residues (His41 and Cys145), as well as the S1 pocket residues (His163, Glu166, and His172) that tightly interact with P1 pyrrolidinone lactam of nirmatrelvir and N3 ligands, were identical in each of the CoV M^pro^ sequences. Amino acids at the S2 and S4 pockets showed slightly more diversity compared to those at S1. The S2 Met49 or Met16 residues become Leu in other β-CoV proteases or Thr in α-CoV proteases (Fig. 1A). The S4 amino acids indicated by the green arrows in Fig. 1A showed even greater diversity compared to those in S2. Although the S2 and S4 amino acids are not completely conserved across different proteases, they still share high sequence similarity. Superposition of the crystal structures of the 12 CoV M^pro^ enzymes illustrated that while they are from different genera and display various levels of sequence identity, they are also structurally similar (Fig. 2A). This is particularly evident within the active site, where the root mean square deviations (RMSDs) of the structures were within 1 Å (Fig. 2B). SARS-CoV-2 and SARS-CoV also shared 100% similarity and identity at the 26 active site residues (Fig. 2B). Overall, we found that both the structure and the sequence of the M^pro^ nirmatrelvir-binding pocket were highly conserved among different CoVs.

### Mutation landscape of M^pro^ from SARS-CoV-2 genomes.

**FIG 3:**
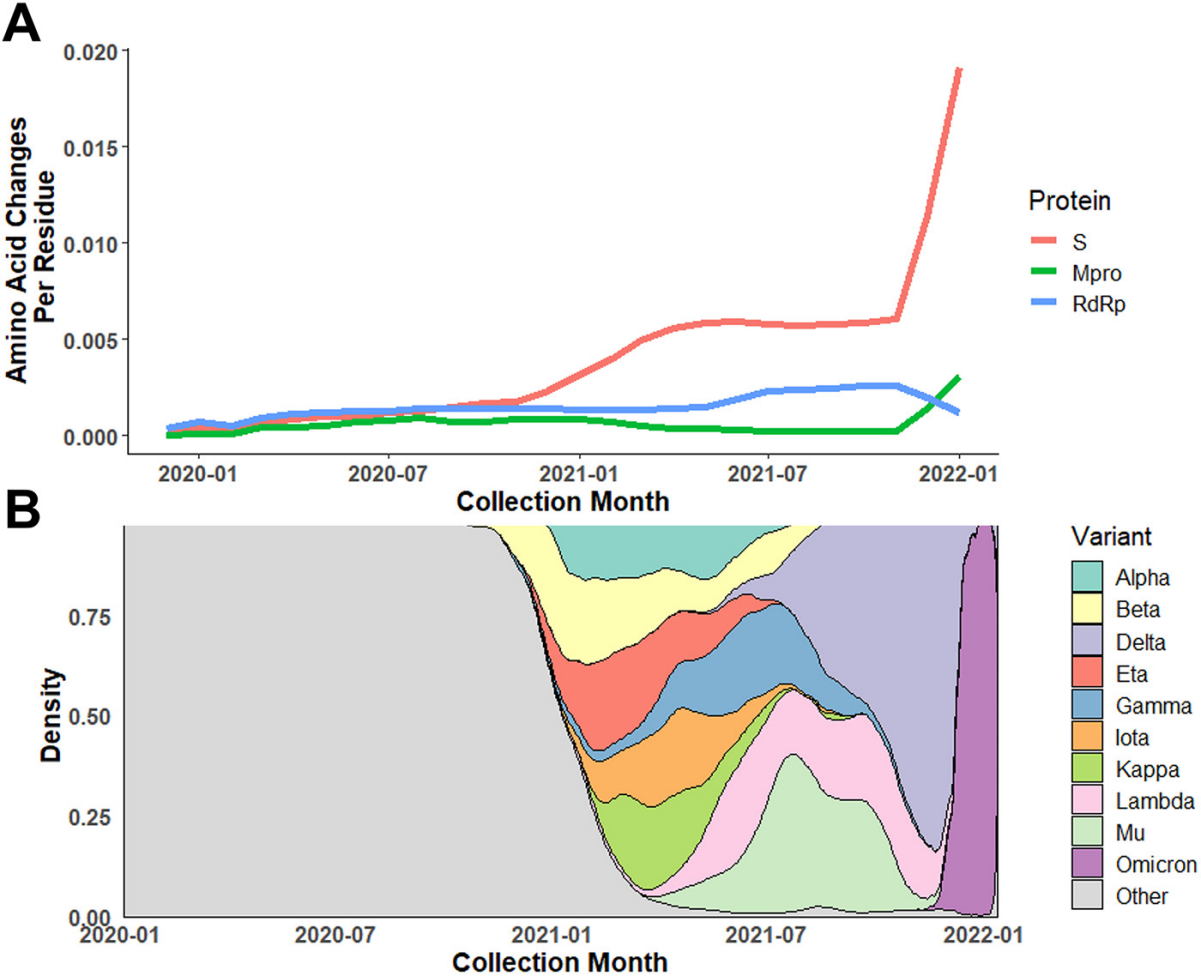
Dynamic change in amino acid mutation rate of M^pro^ compared to Spike protein (S) and RNA-dependent RNA polymerase (RdRp). (A) Average amino acid changes per residue in M^pro^, S protein, and RdRp among isolates collected from January 2020 through January 2022. (B) Relative distribution of variants of concern (VOCs)/variants of interest (VOIs) based on collection date. The rapid rise in amino acid changes found in S protein and M^pro^ near the end of 2021 corresponds to the emergence and takeover of Omicron.

An in-house annotation pipeline was developed to monitor amino acid changes in M^pro^. This pipeline enabled regular retrieval and annotation of the M^pro^ sequence of SARS-CoV-2 genomes obtained from GISAID since the beginning of the pandemic. As of January 14, 2022, 4,892,468 SARS-CoV-2 genomes collected from >250 countries were annotated and examined for mutations in the M^pro^ gene. While ~84% of isolates share the same M^pro^ protein sequence as the reference isolate, ~14,000 unique nucleotide alleles and ~4,800 protein variants have been identified for M^pro^. The nonsynonymous mutation rate (substitution/residue/year) was estimated to be 2.43E−4 for M^pro^, which is lower than RdRp (9.18E−4) and >10-fold lower than S (2.81E−3). The accumulation of amino acid changes per month were plotted for the S, RdRp, and M^pro^ proteins (Fig. 3A). Nonsynonymous changes in M^pro^ remained relatively low and constant compared to RdRp and S prior to December 2021. The first rise of the nonsynonymous mutation rate in the S gene occurred during November through December 2020, which is consistent with emergence of the first two VOCs (Alpha and Beta). Due to the large wave of Omicron isolates collected since the end of 2021, the rate of amino acid changes in both M^pro^ and S has been increasing, with the rise for the S protein being more dramatic compared to M^pro^ and RdRp (Fig. 3B).

The key driver for the evolution of SARS-CoV-2 and numerous VOCs has primarily been adaptive amino acid change observed in the S protein that has enabled evasion of vaccine-elicited immunity or neutralization by MAb therapeutics (26–32). Other than the selection imposed due to its essential function in viral replication and unlike S, M^pro^ has not been subjected to vaccine or antiviral pressure to evolve. It is expected that essential function proteins like M^pro^ are under purifying (negative) selection with a signature nonsynonymous-to-synonymous substitution ratio (*d*_N_/*d*_S_) of less than 1. We conducted a selection analysis using three independent downsampled data sets of three genes: M^pro^, RdRp, and S, with ~80,000 sequences in each data set. The overall mean *d*_N_/*d*_S_ (ω) for M^pro^, RdRp, and S were 0.422 ± 0.009, 0.424 ± 0.011, and 0.550 ± 0.012, respectively. They were all lower than 1, and the *d*_N_/*d*_S_ ratios for M^pro^ and RdRp were lower than that for S, suggesting that M^pro^ and RdRp were under stronger purifying selection compared to S. The nucleotide diversity (π) of M^pro^ was estimated as 6.64E−4, which was lower than that for RdRp (1.02E−3) and S (2.65E−3). Variation of the codon-based *d*_N_/*d*_S_ ratio in M^pro^ was also examined using a Bayesian sliding window model (Fig. S1). Overall, the codon-based *d*_N_/*d*_S_ profile was similar across three independent downsampled data sets. The mean *d*_N_/*d*_S_ ratio across 305 codons in M^pro^ ranged from 0.195 to 0.787. The regions near residues 144 and 289 had lower *d*_N_/*d*_S_ ratios compared to other regions of the protein, indicating that amino acid changes in these regions were not favored and implying that these domains might play critical roles in M^pro^ function.

10.1128/mbio.00869-22.1 FIG S1 Median *d*_N_/*d*_S_ ratio (*ω*) and 95% credibility interval along the M^pro^ gene. The ratio of nonsynonymous-to-synonymous substitutions (*d*_N_/*d*_S_) was calculated using Markov chain Monte Carlo (MCMC) results from three independent subsampling sets of the GISAID sequences (red, blue, and green) to assess the M^pro^ sequence stability. Points represent the *d*_N_/*d*_S_ at each codon, and dotted lines represent the average *d*_N_/*d*_S_ for the gene. The vertical gray lines indicate codons for contact residues. Codons with *d*_N_/*d*_S_ above 1 (dotted line) indicate a greater probability for nonsynonymous mutations, while those below 1 are more conserved and less favored for amino acid changes. The CI alludes to higher *d*_N_/*d*_S_ values around residues 46 and 132 (the second peak aligns with P132H). Download FIG S1, TIF file, 0.2 MB. Copyright © 2022 Lee et al. 2022 Lee et al. https://creativecommons.org/licenses/by/4.0/ This content is distributed under the terms of the Creative Commons Attribution 4.0 International license.

**FIG 4:**
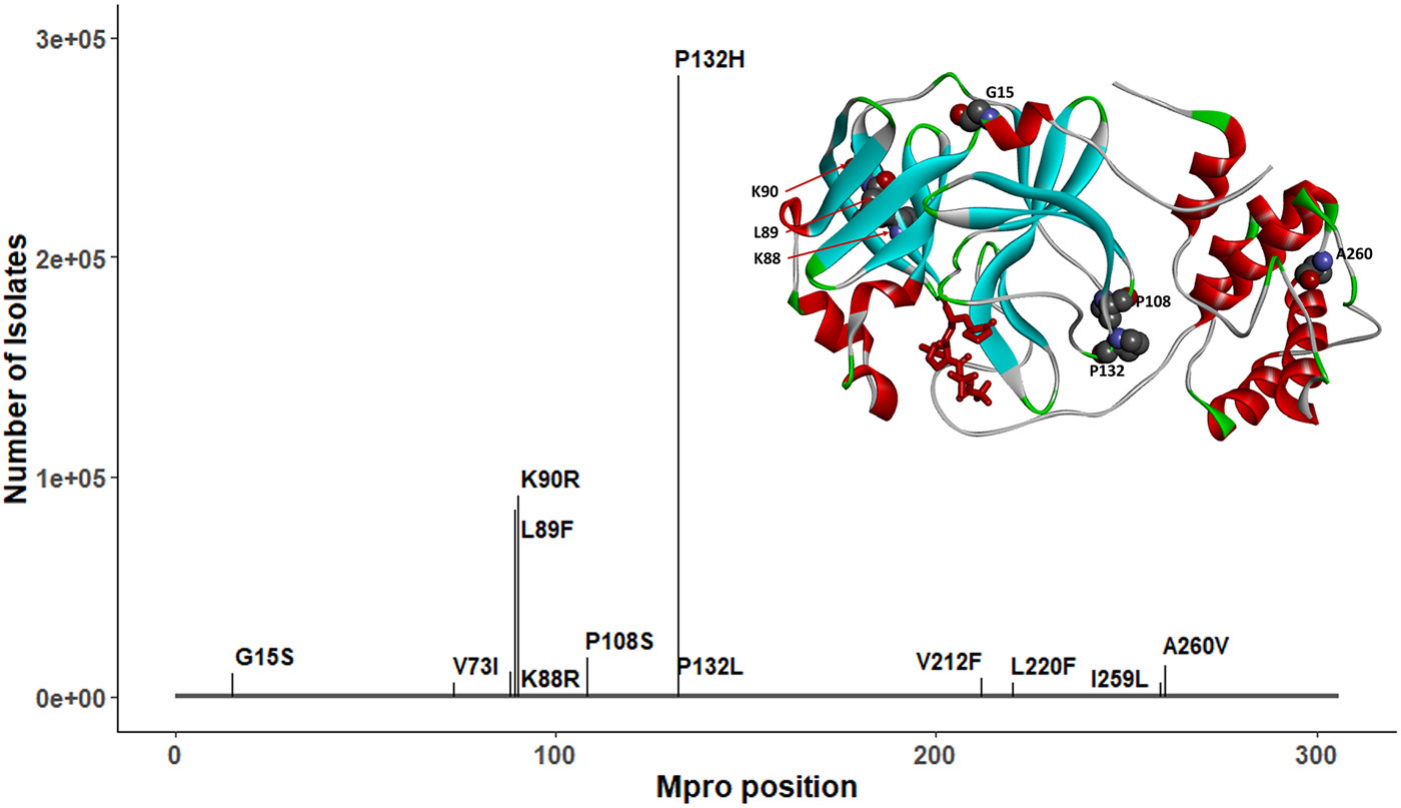
Prevalent mutations in M^pro^ and their position relative to nirmatrelvir binding. Only P132H, characteristic of the Omicron variant, exceeds 100,000 cases, and no residues interact with nirmatrelvir (shown in red). The full geographic and lineage breakdown of these mutations can be found in Table S2.

From examination of the M^pro^ gene across >4.8 million SARS-CoV-2 genomes, the most prevalent mutations (>0.2% mutation frequency) were P132H, K90R, L89F, P108S, A260V, K88R, and G15S (Fig. 4). P132H, with the highest frequency of 6.15%, is exclusively associated with the Omicron VOC (B.1.1.529 or BA.1/2). Prior to the enormous influx of Omicron cases, the frequency of P132H was as low as 0.012%. All prevalent M^pro^ mutations with occurrences >5,000 are listed in Table S2, together with their geographic and genetic lineage distribution. These mutations are associated with different emergent VOCs/variants of interest (VOIs). None of the prevalent mutations mapped to residues critical for nirmatrelvir activity (e.g., proximity of nirmatrelvir-binding pocket as shown in Fig. 1, or dimerization interface, as shown in Fig. S2).

10.1128/mbio.00869-22.2 FIG S2 Position of dimer interface residues in M^pro^. Interface residues were identified as described in Materials and Methods. Each panel A to I shows only one or two protomer contacts for clarity. Residues involved in contact formation are shown in space-filling representations. The protein backbone is shown as solid ribbons: dark blue, domain I of subunit A (residues 1 to 99); light blue, domain I of subunit B; dark green, domain II of subunit A (residues 100 to 182); light green, domain II of subunit B; dark red, domain III (residues 183 to 301) of subunit A; and pink, domain III of subunit B. The figures were rendered with structural coordinates from the PDB ID 7RFS. Download FIG S2, TIF file, 1.1 MB. Copyright © 2022 Lee et al. 2022 Lee et al. https://creativecommons.org/licenses/by/4.0/ This content is distributed under the terms of the Creative Commons Attribution 4.0 International license.

10.1128/mbio.00869-22.4 TABLE S2 Geographic and lineage distribution of the most prevalent M^pro^ mutations (*n *> 5,000). Download Table S2, PDF file, 0.02 MB. Copyright © 2022 Lee et al. 2022 Lee et al. https://creativecommons.org/licenses/by/4.0/ This content is distributed under the terms of the Creative Commons Attribution 4.0 International license.

### Genetic diversity of M^pro^ within variants of concern/interest (VOCs/VOIs).

**FIG 5:**
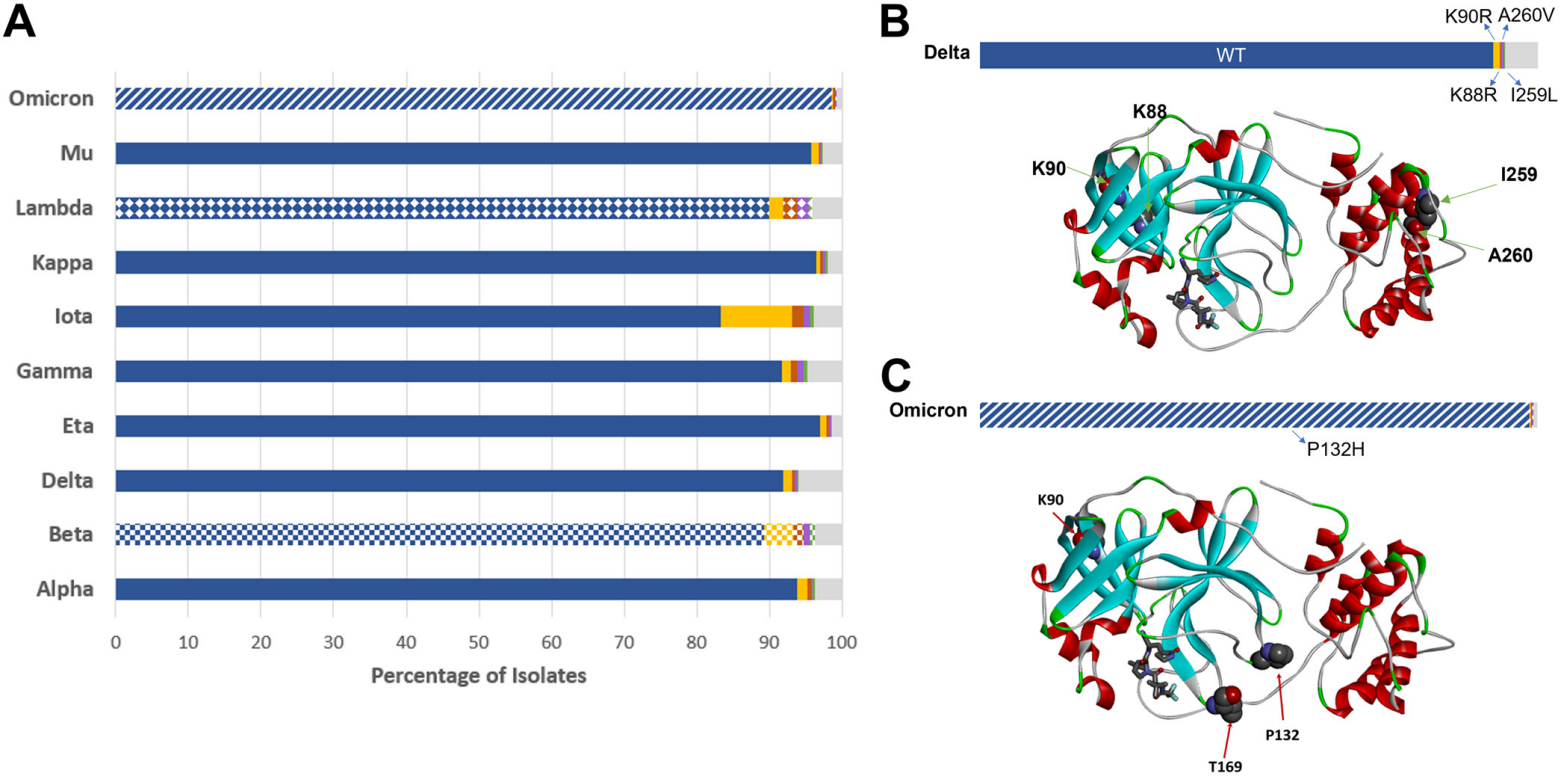
M^pro^ mutations within VOC/VOI populations. (A) The five most prevalent sequences for each lineage are shown as colored bars (blue, gold, red, purple, and green), with the cumulative remaining sequences are in gray. The most prevalent sequence (blue) corresponds to the Wuhan-Hu-1 sequence (wild type [WT]) and is found in all but three lineages. For these remaining lineages (Omicron, Lambda, and Beta), each characteristic nonsynonymous substitution is assigned a pattern: P132H (stripes), G15S (diamonds), and K90R (squares). (B) Relative mutation frequency among Delta variant isolates. The positions of the four most prevalent mutation sites found in this variant (K88, K90, I259, and A260) are shown on the protein structure (WT). (C) Relative mutation frequency among Omicron variant isolates. The positions of the three most prevalent mutation sites (K90, P132, and T169) are shown on the protein structure.

In addition to the five current VOCs, two current VOIs (Lambda and Mu) and three former VOIs (Eta, Iota, and Kappa) have been identified by the WHO (7). In defining SARS-CoV-2 variants, much of the attention is focused on the S protein due to its role in viral biology and selection as a vaccine antigen (8). However, viral lineage assignment takes into account the entire viral genome. It is therefore critical to monitor mutational changes in the viral proteins other than S, including M^pro^, for those VOCs. All M^pro^ protein mutations were retrieved for each individual VOC/VOI. Aside from the Beta, Lambda, and Omicron variants, the majority of isolates from each of the remaining VOCs/VOIs had M^pro^ sequences that were identical to the reference sequence (Wuhan-Hu-1) (Fig. 5A). The P132H mutation was detected in >98% of Omicron isolates, whereas the most prevalent mutations in Lambda and Beta isolates were G15S and K90R, respectively (Fig. 5A). K90R is a conservative substitution and is not expected to induce changes in the three-dimensional structure of the protease, while Gly15 is referred to as a “C′ residue” of the N-terminal α-helix (33, 34), a position with heavy preference for Gly. G15S substitution may lead to a partial decrease in the structural stability of that helix (35), although it is not likely to be detrimental to the overall protein structure.

Prior to the Omicron surge in late 2021, Delta accounted for >90% of SARS-CoV-2 genomes submitted to GISAID (between mid-October and mid-November 2021). To investigate the potential impact of M^pro^ mutations carried by these two major VOCs on inhibitor binding interactions, we mapped the most prevalent mutation sites on the M^pro^ crystal structure with nirmatrelvir for Delta isolates (Lys88, Lys90, Ile259, and Ala260; Fig. 5B) and Omicron isolates (Lys90, Pro132, and Thr169; Fig. 5C). Each of these substitutions is located far from the inhibitor binding site. The most frequent M^pro^ mutation in the Omicron variant, P132H, is unlikely to affect nirmatrelvir inhibitor binding, as the Pro132 residue is located within a flexible turn.

### Genetic diversity at key nirmatrelvir contact residues, cleavage sites, and the dimerization interface of M^pro^.

**FIG 6:**
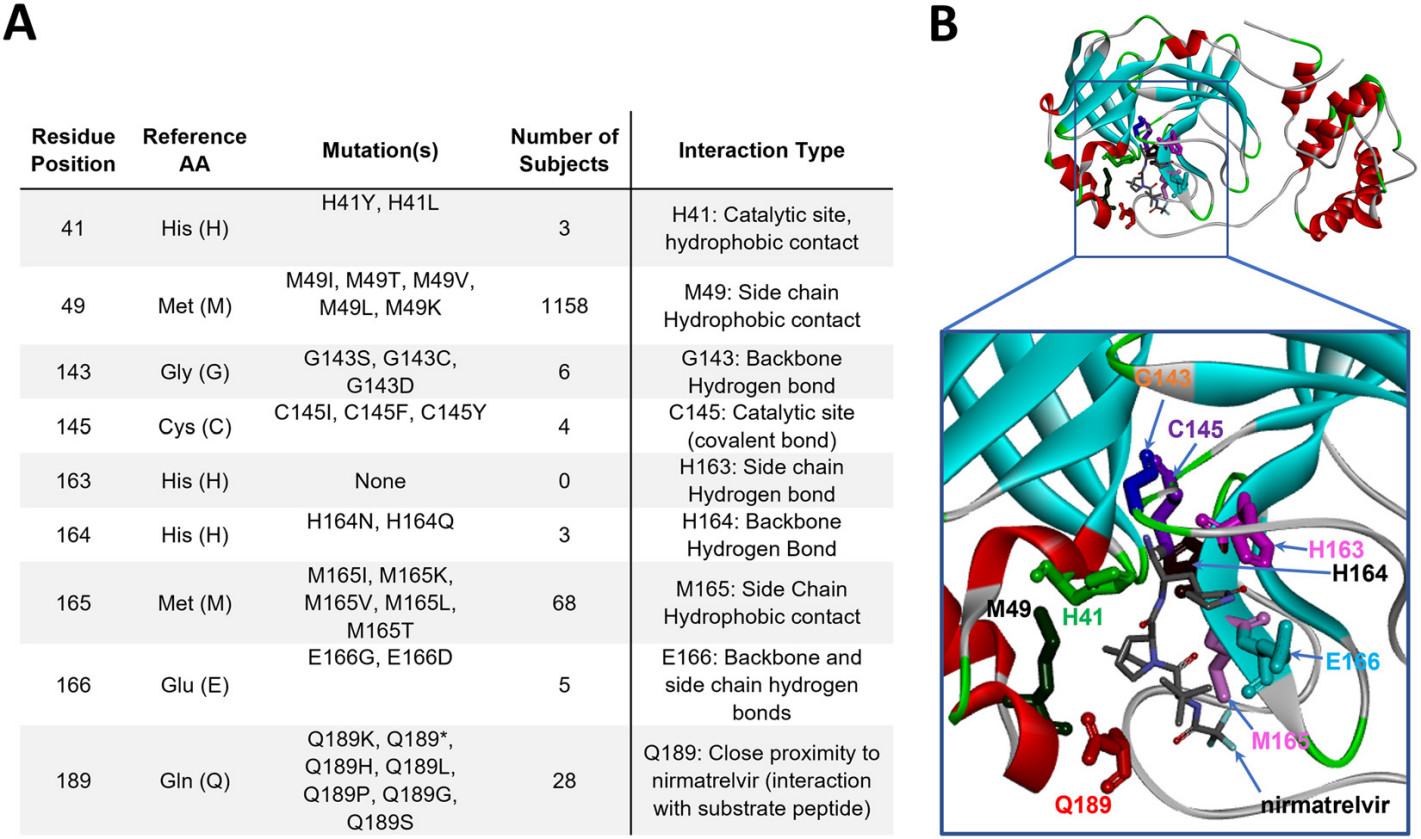
M^pro^ mutation breakdown at nirmatrelvir contact and catalytic residues. (A) Mutations identified at residues directly interacting with nirmatrelvir and/or substrate peptide. (B) Three-dimensional structural model of M^pro^ (PDB ID 7RFS), with residues from panel A highlighted in “stick” representation and shown in individual colors. The protein backbone is shown in ribbon representation. AA, amino acid. Stop codons are donoted as (*).

According to the cocrystal structure of M^pro^ bound to nirmatrelvir reported earlier (11), nine key residues were identified: His41, Met49, Gly143, Cys145, His163, His164, Met165, Glu166, and Gln189 (Fig. 6A). His41 and Cys145 are catalytic residues, while the remaining residues establish direct contacts with nirmatrelvir. Any changes in these residues may affect inhibitor binding. Examination of >4.8 million SARS-CoV-2 genomes illustrated that these nine residues within M^pro^ were highly conserved, with substitution frequencies of <0.028% (Fig. 6B). Among these nine contact residues, one amino acid residue (His163) was not found to be mutated, and five residues (His41, Gly143, Cys145, His164, and Glu166) were extremely conserved with six or fewer isolates identified that carry alternative amino acids. Met49, Met165, and Gln189 had more amino acid changes but still at a frequency of <0.028%.

Another factor that would significantly affect M^pro^ activity and catalytic efficiency is divergence from the consensus substrate recognition sequence, which always contains Gln directly upstream of the cleavage position (position P1). Preceding this (position P2) is a hydrophobic amino acid. At cleavage sites within the SARS-CoV-2 reference isolate Wuhan-Hu-1, this is most commonly Leu, but some substrates contain Phe or Val at this position. The residue directly downstream of the cleavage site (P1′) is generally Ser or Ala, with Asn observed in one case. Other residues further from the cleavage position are less well conserved across target sites within SARS-CoV-2. The sequence of M^pro^ cleavage sites and neighboring residues in the reference isolate Wuhan-Hu-1 (NC_045512.2) are listed in Table S3.

10.1128/mbio.00869-22.5 TABLE S3 M^pro^ cleavage sites and coordinates across ORF1ab. Download Table S3, PDF file, 0.1 MB. Copyright © 2022 Lee et al. 2022 Lee et al. https://creativecommons.org/licenses/by/4.0/ This content is distributed under the terms of the Creative Commons Attribution 4.0 International license.

We investigated the mutation frequency of >4.8 million isolates at the 11 M^pro^ substrate cleavage sites and neighboring residues along ORF1ab to assess sequence conservation. In total, 445 unique amino acid changes were identified within five residues of the cleavage sites (Table S4). Despite being the most conserved amino acid among the 11 recognition sites on the Wuhan-Hu-1 reference, the P1 Gln was not the most conserved residue among the examined isolates. Rather, both the P2 and P1′ positions had fewer mutations overall. In total, 7,282 instances of substitution at position P1 were observed with >98% of those cases being Gln to His (Table S4). Over 5,000 cases of this mutation were at the Nsp8-Nsp9 junction, with no more than 1,000 changes from the Gln consensus at P1 detected at any of the other 10 cleavage sites (Table S4). Consistent with the role of a hydrophobic residue at P2, ~95% of the 4,019 amino acid changes at this position were to Leu, Ile, Val, and Phe. Meanwhile, of 5,914 mutations at P1′, the most common was Ala to Ser, the two amino acids generally found at this position across cleavage sites. Aside from the downstream P3′ and P5′ positions, all other positions within five residues of the cleavage site had a greater incidence of mutation than positions P1, P2, and P1′ (Table S4).

10.1128/mbio.00869-22.6 TABLE S4 Mutation frequency at M^pro^ cleavage sites and neighboring residues. Download Table S4, XLSX file, 0.03 MB. Copyright © 2022 Lee et al. 2022 Lee et al. https://creativecommons.org/licenses/by/4.0/ This content is distributed under the terms of the Creative Commons Attribution 4.0 International license.

M^pro^ dimerization is critical for enzyme function, and the strength of the interprotomer contact can directly affect protease activity (36–39). Given the importance of dimerization, we performed analysis of amino acid residue conservation at this interface (Table 1). That interface is formed by the N-terminal tail of each protomer inserted between the two subunits of the enzyme, with many residues forming a complex network of interactions. Seventeen residues predicted to impact dimerization through interaction with one another were identified (Fig. S2). As predicted from the dimerization requirement for enzyme activity, these residues were also highly conserved with a mutation frequency of <0.11% across the >4.8 million SARS-CoV-2 genomes examined (Table 1). No substitutions were detected at Glu290, and six other residues (Glu14, Tyr126, Ser139, Glu166, Leu286, and Gln299) displayed extreme conservation with less than six instances of alternative amino acids. Residue Ala285 had the largest diversity among amino acids within the dimerization motif, although still at a frequency of only ~0.03%.

**TABLE 1:** Mutation breakdown at M^pro^ dimerization interface residues

Residue position	Reference AA^*a*^	Mutations	No. of subjects	No. of countries	No. of lineages	Countries	Lineages	Characteristics
1	Ser (S)	S1C, S1G, S1N	135	4	6	UK (90.37%), Australia (6.67%)	B.1.617.2 (88.89%), D.2 (6.67%)	Side chain hydrogen bond to the side chain of E166
4	Arg (R)	R4K, R4I, R4S, R4G	593	27	47	USA (34.74%), Poland (20.24%), Germany (11.3%), UK (7.93%), Sweden (6.58%)	B.1.617.2 (20.41%), AY.122 (18.03%), AY.100 (10.71%), AY.25.1 (6.8%), B.1.1.7 (5.95%), B.1.177 (5.78%)	Salt bridge to E290
6	Met (M)	M6L, M6I, M6T, M6V, M6R	158	18	35	USA (39.24%), UK (18.99%), France (18.35%), Switzerland (5.06%)	B.1 (9.49%), B.1.160 (9.49%), BA.1 (9.49%), AY.4 (8.86%), B.1.1.7 (8.23%), B.1.617.2 (6.96%), AY.44 (6.33%), AY.12 (5.7%)	van der Waals/hydrophobic interaction with the side chain of Y126
7	Ala (A)	A7V, A7T, A7S, A7G, A7P	1,053	42	79	USA (63.06%), Mexico (6.74%), UK (6.55%)	AY.25 (21.51%), AY.44 (14.44%), B.1.617.2 (14.44%), B.1.632 (6.31%), AY.4 (5.83%), B.1.1.7 (5.26%)	van der Waals/hydrophobic interaction with the side chain of V125
9	Pro (P)	P9S	45	2	3	South Korea (97.78%)	B.1.497 (95.56%)	van der Waals/hydrophobic interaction with the side chain of P122
12	Lys (K)	K12R, K12N	338	13	23	USA (71.01%), UK (21.3%)	B.1.617.2 (45.24%), AY.103 (20.54%), AY.4 (11.61%), B.1.1.7 (8.63%)	Electrostatic interaction with the side chain of E14^*b*^
14	Glu (E)	E14D, E14*^c^	6	3	4	USA (66.67%), Sweden (16.67%), UK (16.67%)	B.1.617.2 (50.0%), AY.100 (16.67%), AY.4 (16.67%), AY.9.1 (16.67%)	Side chain hydrogen bond to backbone amide of G11; electrostatic interaction with the side chain of K12^*a*^
122	Pro (P)	P122S, P122L, P122I, P122A	121	20	31	UK (41.32%), USA (25.62%), France (9.92%)	B.1.617.2 (23.14%), AY.4 (20.66%), AY.118 (6.61%), B.1.1.7 (5.79%)	van der Waals/hydrophobic interaction with the side chain of P9
125	Val (V)	V125I, V125A, V125L	361	26	40	UK (34.9%), USA (29.36%), Canada (9.42%), Germany (6.65%)	AY.4 (24.93%), B.1.617.2 (21.05%), AY.25 (9.42%), AY.98 (6.37%)	van der Waals/hydrophobic interaction with the side chain of A7
126	Tyr (Y)	Y126C, Y126P	4	2	4	Turkey (50.0%), USA (50.0%)	B.1.1.7 (25.0%), B.1.177.86 (25.0%), B.1.351 (25.0%), B.1.400 (25.0%)	van der Waals/hydrophobic interaction with the side chain of M6
139	Ser (S)	S139A, S139T	6	2	2	UK (66.67%), USA (33.33%)	BA.1 (66.67%), B.1.399 (33.33%)	Side chain hydrogen bond to the side chain of Q299
166	Glu (E)	E166G, E166D	5	4	5	USA (40.0%), Finland (20.0%), Nigeria (20.0%), Switzerland (20.0%)	AY.107 (20.0%), AY.39 (20.0%), B.1.177.23 (20.0%), B.1.525 (20.0%), B.1.617.2 (20.0%)	Side chain hydrogen bond to the side chain of S1
285	Ala (A)	A285V, A285P, A285T, A285D, A285S, A285E, A285G	1,426	57	115	USA (25.74%), Switzerland (18.37%), UK (10.87%), Brazil (6.1%)	B.1.1.29 (17.21%), B.1.617.2 (11.8%), B.1.1.7 (7.37%), AY.4 (7.09%)	van der Waals/hydrophobic interaction with the side chains of A285 and L286
286	Leu (L)	L286I, L286F	6	4	5	USA (50.0%), Egypt (16.67%), Netherlands (16.67%), UK (16.67%)	B.1.2 (33.33%), AY.3 (16.67%), AY.4.2 (16.67%), B.1 (16.67%), B.1.617.2 (16.67%)	van der Waals/hydrophobic interaction with the side chain of A285
290	Glu (E)		0	–^*d*^	–	–	–	Salt bridge to R4
298	Arg (R)	R298K, R298G, R298I, R298S, R298T	582	34	52	UK (58.25%), USA (20.96%)	AY.4 (43.47%), B.1.617.2 (24.4%)	Side chain hydrogen bond to the backbone of S123
299	Gln (Q)	Q299H	3	1	1	Nigeria (100.0%)	B.1.1.7 (100.0%)	Side chain hydrogen bond to the side chain of S139

a AA, amino acid.

b Side chains of K12 and E14 are over 5 Å apart. Hence, an actual salt bridge is not likely to form, although a relatively weak ionic attraction cannot be ruled out.

c Stop codons are denoted with (*).

d Dashes (-) indicate that no data was available at the time of this study.

## DISCUSSION

For the first time, pathogen population genomics has been applied in real time to track emerging SARS-CoV-2 variants and guide the public health response to the pandemic (18). We have developed an analysis workflow to routinely annotate M^pro^ sequences and other regions of interest through genotypic surveillance. Utilizing a data set of nearly 4.9 million SARS-CoV-2 genomes in GISAID, our analysis of the M^pro^ mutational landscape revealed that pre-existing mutations at residues interacting with nirmatrelvir, as well as at the cleavage junctions and dimerization interface, that may contribute to drug resistance were rare. The distances of the nine contact/catalytic sites to nirmatrelvir are all less than 4 Å. Notably, among the residues with key ligand interaction, only two residues (Met49 and Met165) were more frequently changed compared to others with a hydrogen bond or near the catalytic active site. Met49 and Met165 make side chain hydrophobic contacts to the inhibitor, especially for residue Met49, which has the largest number of occurrences (*n = *1,098) among all close contact sites examined herein. It is likely that Ile at this position is acceptable since Met and Ile side chains are similar in shape and polarity, as discussed previously (40).

The considerable degree of structural similarity at the M^pro^ nirmatrelvir-binding pocket across the different groups of CoVs may explain the consistent broad biochemical potency of nirmatrelvir against multiple CoVs, including SARS-CoV, Middle Eastern respiratory syndrome (MERS)-CoV, murine hepatitis virus (MHV), OC43, HKU1, 229E, NL63, and IBV proteases, as reported previously (11). In addition to the residues forming nirmatrelvir-binding sites, variation in residues at the M^pro^ dimer interface was also monitored, as self-association is critical for protease activity. Although not all residues at the interface have been proven to be functionally important, it is conceivable that amino acid substitutions at positions that are spatially close to each other may introduce favorable or unfavorable interactions. In turn, this could result in changes in subunit association and, correspondingly, an impact on enzyme activity and/or nirmatrelvir binding.

Our selection analysis on M^pro^ demonstrated that the protein is under strong purifying selection with a nonsynonymous-to-synonymous mutation ratio (*d*_N_/*d*_S_) of less than 1. This is consistent with previous observations (41). However, mutations in M^pro^ could populate quickly due to the “founder effect,” when a new variant (VOC/VOI) emerges, becomes dominant in a population, and reduces genetic variation. For example, the ancestral Omicron variant always carried the P132H mutation in M^pro^. In late 2021, P132H became the most prevalent M^pro^ mutation with its frequency rapidly jumping from 0.012 to 6.15% after the Omicron surge, although this mutation does not necessarily offer any selective advantage on viral fitness or alter inhibitor potency of nirmatrelvir (42). As expected, nirmatrelvir maintains antiviral activity against all five VOCs and two VOIs in M^pro^, including Omicron, Beta, and Lambda, which carry the P132H, K90R, and G15S mutations, respectively (43–47). This may change with widespread use of nirmatrelvir, which, not unlike the antibodies against the S protein, may exert selective pressure on its target, leading to a reduction of potency. We anticipate, however, that this possibility would be mitigated by the key features in the chemical design and the use of Paxlovid, such as maintaining structural similarity with the native substrate of M^pro^ (11), a short treatment window (5 days), and a low dose of ritonavir (100 mg) (48).

It is important to note that although this analysis provides data on what is currently circulating, this is not a prevalence-based analysis and is biased by geographic regions that are routinely sequencing isolates, with ~55% of submitted viral genomes originating from the United Kingdom and the United States. Another caveat of using GISAID data sets is that only consensus genome sequences are available. Potential emerging resistant mutations usually have low frequency (minor allele) within viral quasispecies and will not be uncovered from assembled genomic contigs. The presence of artifacts in assembled sequencing data are also expected due to inevitable errors in the sequencing process. While GISAID has implemented internal checks to flag potential errors in submitted assemblies, this does not eliminate the potential risk of misinterpreting artifacts as mutations. Nonetheless, the vast number of sequences available for analysis (>7 million SARS-CoV-2 genomes as of January 14, 2022) proved valuable in providing a comprehensive picture of the mutational landscape of M^pro^.

At present, SARS-CoV-2 continues to represent a global health threat as new variants emerge. It is essential to continue tracking M^pro^ mutations in global viral isolates, especially since nirmatrelvir, the active protease inhibitor in Paxlovid, is expected to become a widely accessible COVID-19 treatment option. However, at present, nirmatrelvir has yet to be deployed on a mass scale. Following FDA approval of remdesivir, its widespread usage in hospitals for the first year and a half of the COVID-19 pandemic has permitted analyses of known resistance mutations in viral isolates under remdesivir selection (49). Therefore, as more sampled viral isolates undergo nirmatrelvir selection and as more sequences become available in GISAID, our analysis workflow is prepared to detect the emergence of potential escape mutations. Moving forward, genomic surveillance of M^pro^ will be needed to continuously assess risk for antiviral resistance, specifically in the context of Paxlovid treatment of patients with active SARS-CoV-2 infection. In addition, mutation analysis of viral sequence data for participants enrolled in Pfizer Paxlovid clinical study (EPIC-HR), a phase 2/3 randomized placebo-controlled trial in subjects with laboratory-confirmed diagnosis of SARS-CoV-2 infection, is currently ongoing.

In conclusion, the results of our extensive sequence analysis across nearly 4.9 million global SARS-CoV-2 isolates, including the recently emerged Omicron variant, highlight the high genetic conservation of the M^pro^ protein. We have built a robust workflow to monitor mutational changes in nirmatrelvir contact residues, polymorphism of cleavage and dimerization sites, and M^pro^ structural differences between SARS-CoV-2 and other CoVs. As new antiviral monotherapies against SARS-CoV-2 are introduced in the coming months, the potential for drug resistance is a serious concern. The genetic stability and structural conservation of M^pro^ observed over time in SARS-CoV-2 variants suggests a minimal global risk of pre-existing resistance to nirmatrelvir. An established system to surveil real-world genomic data for emerging resistant mutations is critical as the SARS-CoV-2 virus continues to evolve under the various selective pressures imposed by humans.

## MATERIALS AND METHODS

### Structural comparison of M^pro^ from different CoVs.

The crystal structures of M^pro^ from multiple CoVs have been reported previously in either apo or inhibitor-bound form (21–24). The Protein Data Bank structures that were selected as representatives for analysis are listed in Table S1 (*n = *12). The active site amino acids are defined as those within 4.5 Å of the common ligand PRD_002214. The chain A of 11 M^pro^ proteins were superimposed on the SARS-CoV-2 M^pro^ protein complexed with nirmatrelvir (PDB ID 7RFW) based on the carbon-α (Cα) of the 26 amino acids. The superposition of images was generated using the Molecular Operating Environment (MOE) software platform (version 2020.09, Chemical Computing Group ULC, Montreal, Quebec, Canada). The RMSD was also calculated based on the 26 Cα atoms.

### SARS-CoV-2 genomes and M^pro^ annotation pipeline.

Genome sequences and patient metadata for ~4.9 million isolates were obtained from the GISAID (17) EpiCoV database (www.epicov.org) through January 14, 2022. The genomes were quality filtered: incomplete genomes <29,000 nucleotides in length and/or containing >5% ambiguous nucleotides (Ns) were excluded. Sequences, collection dates, countries of origin, and lineage assignments were deposited to an internal database, BIGSdb (50).

M^pro^ nucleotide sequences were obtained using BLASTN alignment (51) to the reference SARS-CoV-2 genome (NC_045512.2, isolate Wuhan-Hu-1) (52). Sequences with less than 90% alignment or containing ambiguous bases were excluded from further analysis. Nucleotide alleles were translated to amino acid sequences, and nonsynonymous polymorphisms were called through pairwise alignment to the reference M^pro^ amino acid sequence of the Wuhan-Hu-1 isolate. The protein sequences were assigned unique IDs linked to the respective viral genomes in BIGSdb.

### Nonsynonymous mutation rate calculation.

A list of mutation fingerprints (MFs) was downloaded from the COVID-19 Virus Mutation Tracker (CoVMT) (53) (https://www.cbrc.kaust.edu.sa/covmt/). A MF was defined as the specific set of mutations shared by a group of genomic isolates from GISAID. The MF list is regularly updated and maintained by the CoVMT team. An *ad hoc* script was written to calculate the number of nonsynonymous mutations occurring on the M^pro^, RdRp, and S genes per month. The amino acid mutation rate for each gene was then calculated and plotted by month of sample collection.

### Nucleotide diversity and *d*_N_/*d*_S_ selection analysis.

Because selection analysis tools are computationally intensive, the genome data set retrieved from GISAID was randomly downsampled to a manageable subset (~80,000) using the Nextstrain Augur pipeline (54) with a maximum of 100,000 sequences equally sampled by geographic region and month from December 1, 2020, through January 1, 2022. Three downsampled subsets of SARS-CoV-2 genomes were independently generated. Each subset of genomes was then aligned to the reference genome (Wuhan-Hu-1) using MAFFT (55) (with a –6-mer pair flag for rapid alignment of large numbers of closely related viral genomes). M^pro^, RdRp, and S genes were extracted from the genome-wide alignments. To prepare for selection analysis, sequences with entries of N or with deletions (noted with hyphens) were filtered out for M^pro^ and RdRp genes. Any non-ATGC characters or STOP codons were replaced with triplet of hyphens, and the sequences were retained in the data set. As the S gene has many deletions, to maintain a comparable number of sequences, the sequences with deletions were not filtered out, and instead, those with non–in-frame deletions were replaced with in-frame deletions. This was performed by converting each partial indel to an indel (e.g., converting -AC to ---). Overall nucleotide diversity was inferred using MEGA X (56). The ratio of nonsynonymous-to-synonymous mutations (*d*_N_/*d*_S_ or ω) was inferred using GenomegaMap (57) (Bayesian sliding window model) with the transition:transversion ratio (κ) of 1.0 and nucleotide diversity (θ) of 0.17. Two independent Markov chain Monte Carlo (MCMC) analyses were run at 500,000 iterations each. The runs were compared for convergence, and the resulting *d*_N_/*d*_S_ values were determined using RStudio (version 1.1.383). The average of *d*_N_/*d*_S_ from three downsampled data sets were used for our selection analysis.

### SARS-CoV-2 intralineage M^pro^ diversity analysis.

The five most prevalent M^pro^ protein sequences among GISAID isolates were retrieved from BIGSdb for each VOI or VOC. Any polymorphisms among these sequences were determined from the prior alignments. The total instances of each mutation were then obtained based on sequence prevalence within each SARS-CoV-2 lineage.

### Structural analysis of the M^pro^ dimer interface.

Residues involved in stabilization of the M^pro^ dimer interface were identified from the structure of the dimeric SARS-CoV-2 M^pro^ (PDB ID 7RFR) (11) (Table 1). Interprotomer contacts were initially identified using the Biovia Discovery Studio Visualizer (version 4.5, Dassault Systèmes) and then manually inspected to confirm. All structural models of the M^pro^ protein were rendered using the Biovia Discovery Studio Visualizer software.

### Data availability.

All viral genome sequences analyzed herein were obtained from the GISAID public database (17) (www.gisaid.org). These sequences represented accessions for samples deposited between January 10, 2020, and January 14, 2022. The accession numbers total in the millions.
