# Supplementary material for: Genetic Surveillance of SARS-CoV-2 Mpro Reveals High Sequence and Structural Conservation Prior to the Introduction of Protease Inhibitor Paxlovid
Source: mBio. 2022 Jul 13;13(4):e00869-22. doi: 10.1128/mbio.00869-22 (PMC9426535; doi:10.1128/mbio.00869-22)
Supplement: TABLE S1 [file mbio.00869-22-s0003.pdf]

Table S1. List of CoV protease structures in the homology analysis.

Protein	Group	Pdb ID	Ligand
SARS-CoV-2 main protease	beta	7RFW	PF-07311332
SARS-CoV-2 main protease	beta	6LU7	PRD_002214
SARS-CoV-1 main protease	beta	2AMQ	PRD_002214
MERS-CoV	beta	4RSP	PRD_002174
MHV (Murine hepatitis virus)	beta	6JIJ	PRD_002214
HKU1	beta	3D23	PRD_002214
HKU4 (Tylonycteris bat coronavirus)	beta	4YOG	4F5
229E	alpha	2ZU2	apo
PEDV (Porcine epidemic diarrhea virus)	alpha	5GWZ	PRD_002214
FIPV (Feline infectious peritonitis virus)	alpha	5EU8	PRD_002214
NL63	alpha	5GWY	PRD_002214
IBV (infectious bronchitis virus)	gamma	2Q6F	PRD_002214
