# Supplementary figures and images for: Genetic Surveillance of SARS-CoV-2 Mpro Reveals High Sequence and Structural Conservation Prior to the Introduction of Protease Inhibitor Paxlovid
Source: mBio. 2022 Jul 13;13(4):e00869-22. doi: 10.1128/mbio.00869-22 (PMC9426535; doi:10.1128/mbio.00869-22)

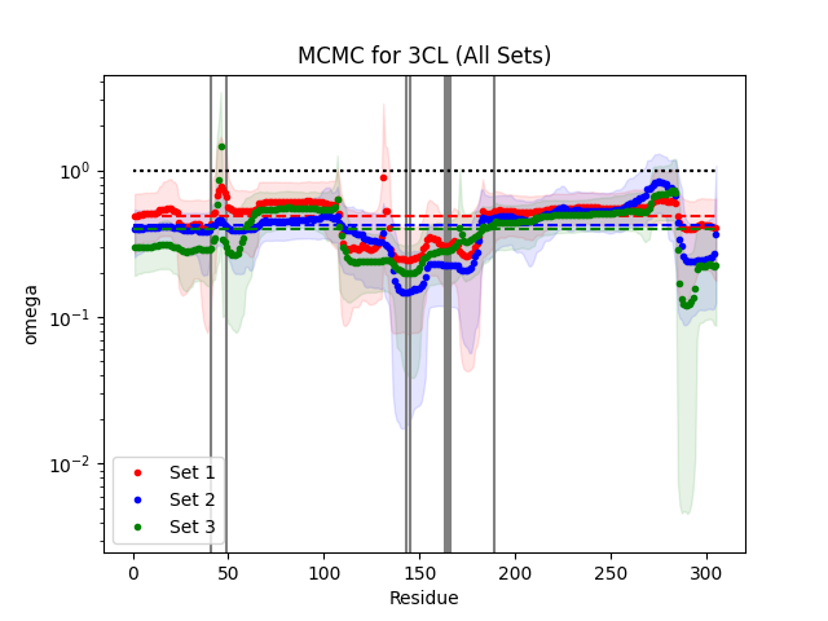

Supplement: FIG S1 [file mbio.00869-22-s0001.tif]

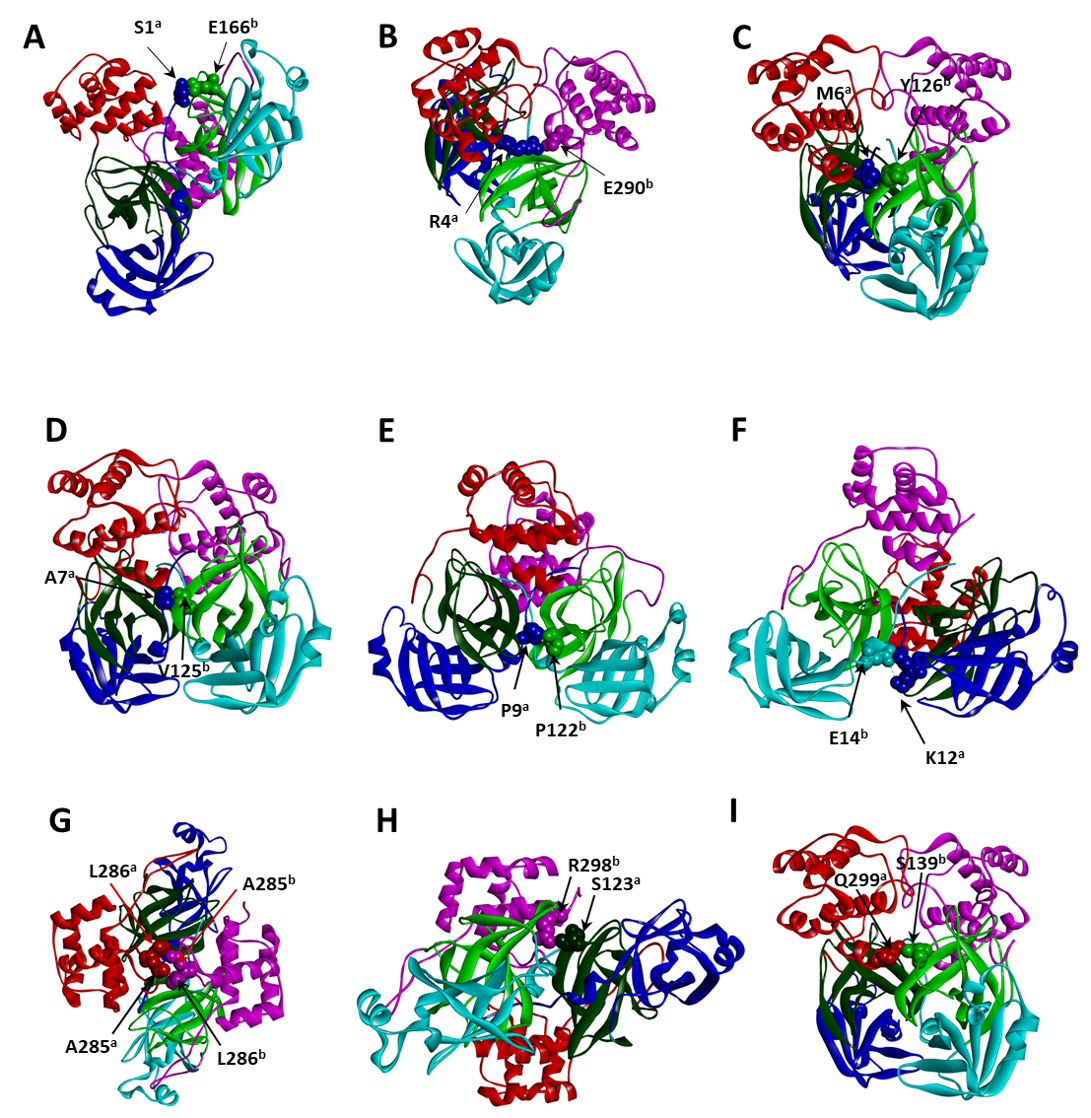

Supplement: FIG S2 [file mbio.00869-22-s0002.tif]
