# Supplementary material for: Genetic Surveillance of SARS-CoV-2 Mpro Reveals High Sequence and Structural Conservation Prior to the Introduction of Protease Inhibitor Paxlovid
Source: mBio. 2022 Jul 13;13(4):e00869-22. doi: 10.1128/mbio.00869-22 (PMC9426535; doi:10.1128/mbio.00869-22)
Supplement: TABLE S2 [file mbio.00869-22-s0004.pdf]

Table S2. Geographic and lineage distribution of the most prevalent M^{pro} mutations (*n* > 5,000).

Mutations	Population Frequency (%)	Number of Countries	Number of Lineages	Top Countries	Top Lineages
P132H	6.15	97	54	UK (44.6%), US (32%), Denmark (6.7%)	BA.1 (98.1%)
K90R	1.987	165	549	US (26.3%), UK (10.2%), Denmark (9.8%)	B.1.351 (23.1%), B.1.617.2 (15%), B.1.1.7 (12.1%), AY.4 (10.4%)
L89F	1.857	90	149	US (92.7%)	B.1.2 (91.3%)
P108S	0.381	84	247	Japan (54.9%), US (15%), Switzerland (7.2%), UK (5.7%)	B.1.1.284 (52.9%), B.1.1.7 (15.1%), B.1.617.2 (7.9%)
A260V	0.312	90	249	US (29.9%), UK (22.3%), Germany (8.4%), Japan (5.8%)	B.1.617.2 (14.9%), AY.4 (14%), B.1.1.7 (12.3%), AY.44 (11.9%), AY.122 (10.4%), B.1.177 (5.2%)
K88R	0.238	62	149	UK (78.3%), US (7%)	AY.4 (75.7%), B.1.617.2 (5.3)
G15S	0.23	108	124	UK (26.2%), Peru (10.5%), US (8.1%)	B.1.1.1 (43.3%), C.37 (10%), C.36 (8.4%), C.36.3 (5.7%)
V212F	0.174	39	62	Denmark (86.3%)	AY.4 (74.8%), AY.4.6 (12.1%), B.1.367 (8.6%)
I259L	0.137	17	46	US (84.7%), UK (13.9%)	AY.3 (95.8%)
L220F	0.134	49	104	US (49.2%), Denmark (37.5%)	AY.25 (31.1%), B.1.617.2 (24.1%), AY.122 (23%)
P132L	0.133	72	174	US (19.7%), UK (16.8%), Germany (14.2%), Netherlands (12.6%), Denmark (12.2%)	B.1.617.2 (18.4%), AY.9 (12.8%), AY.66 (8.1%), AY.9.2 (8%), AY.4 (7.8%), B.1.1.7 (5%)
V73I	0.132	31	55	US (98%)	B.1.617.2 (58.6%), AY.118 (24.2%), AY.4 (8.4%)
V157L	0.128	59	126	Denmark (52.1%), US (11.5%), UK (9.6%), Canada (8.9%), Germany (5.7%)	AY.43 (52.6%), AY.4 (8.8%), B.1.1 (8.2%), AY.4.3 (5.4%)
L75F	0.116	78	203	US (36.9%), UK (9.6%), Switzerland (7.2%), Germany (6.8%)	B.1.1.7 (35.6%), B.1.617.2 (9.6%), AY.3 (6.9%), B.1.526 (6.2%)
T196M	0.112	52	110	US (83%)	B.1.429 (37.4%), AY.44 (22.6%), B.1.234 (12%), B.1.617.2 (8.2%)
A191V	0.111	82	245	US (38.8%), UK (30.6%)	AY.4 (13.6%), B.1.617.2 (9.9%), B.1.177.20 (6.2%), AY.103 (5.9%), B.1.1.7 (5.8%), AY.25 (5%)
A129V	0.11	71	177	UK (44.9%), US (28.2%)	AY.4 (33.4%), B.1.617.2 (19.1%), AY.25 (5.6%), B.1.1.7 (5.1%)
