# Supplementary material for: Genetic Surveillance of SARS-CoV-2 Mpro Reveals High Sequence and Structural Conservation Prior to the Introduction of Protease Inhibitor Paxlovid
Source: mBio. 2022 Jul 13;13(4):e00869-22. doi: 10.1128/mbio.00869-22 (PMC9426535; doi:10.1128/mbio.00869-22)
Supplement: TABLE S3 [file mbio.00869-22-s0005.pdf]

Table S3. M^{pro} cleavage sites and coordinates across ORF1ab.[a]

Proteins		Cleavage Sites	ORF1ab Coordinates
ORF1	ORF2		
nsp4	nsp5	SAVL**Q**↓SGFRK	3259 .. 3268
nsp5	nsp6	GVTF**Q**↓SAVKR	3565 .. 3574
nsp6	nsp7	VATV**Q**↓SKMSD	3855 .. 3864
nsp7	nsp8	RATL**Q**↓AIASE	3938 .. 3947
nsp8	nsp9	AVKL**Q**↓NNELS	4136 .. 4145
nsp9	nsp10	TVRL**Q**↓AGNAT	4249 .. 4258
nsp10	nsp11/12	EPML**Q**↓SADAQ	4388 .. 4397
nsp12	nsp13	HTVL**Q**↓AVGAC	5320 .. 5329
nsp13	nsp14	VATL**Q**↓AENVT	5921 .. 5930
nsp14	nsp15	FTRL**Q**↓SLENV	6448 .. 6457
snp15	nsp16	YPKL**Q**↓SSQAW	6794 .. 6803

[a] from the SARS-CoV-2 reference sequence (NC_045512.2, isolate Wuhan-hu-1)
